# Targeting inflammation to influence mood following spinal cord injury: a randomized clinical trial

**DOI:** 10.1186/s12974-015-0425-2

**Published:** 2015-11-06

**Authors:** David J. Allison, David S. Ditor

**Affiliations:** Department of Kinesiology, Faculty of Applied Health Science, Brock University, 1812 Sir Isaac Brock Way, St. Catharines, ON L2S 3A1 Canada; Brock-Niagara Centre for Health and Well-Being, St. Catharines, ON L2T 1W4 Canada

**Keywords:** Depression, Mood, Spinal cord injury, Inflammation, IL-1β, Anti-inflammatory diet

## Abstract

**Background:**

The purpose of the present study was to examine the efficacy of targeting inflammation as a means of improving mood following spinal cord injury (SCI) and explore the potential mechanisms of action.

**Methods:**

The study was a randomized, parallel-group, controlled, clinical trial (NCT02099890) whereby 20 participants with varying levels and severities of SCI were randomized (3:2) to either the treatment group, consisting of a 12-week anti-inflammatory diet, or control group. Outcome measures were assessed at baseline, 1 and 3 months, and consisted of CES-D scores of depression, markers of inflammation as assessed by various pro- and anti-inflammatory cytokines and several amino acids related to depression.

**Results:**

A significant group × time interaction was found for CES-D (Center for Epidemiologic studies Depression Scale) score (*p* = 0.01), the TRP/LNAA (tryptophan/large neutral amino acid) ratio (*p* = 0.04), the composite score of pro-inflammatory cytokines (*p* = 0.04), IL-1β (interleukin-1 beta) (*p* = 0.04), and IFN-γ (interferon gamma) (*p* = 0.03). Pearson’s *r* correlation showed significance between the ∆IL-1β and both the ∆CES-D score (*r* = 0.740, *p* < 0.01) and the ∆KYN/TRP (kynurenine/tryptophan) ratio (*r* = 0.536, *p* = 0.02). The ∆KYN/TRP ratio was also significantly correlated with the ∆CES-D score (*r* = 0.586, *p* = 0.01). Mediation analysis showed that the relationship between the ∆KYN/TRP ratio and the ∆CES-D score was mediated by the ∆IL-1β. Subgroup analysis showed that participants with high CES-D scores had significantly higher concentrations of IL-1β, and all correlations were maintained or strengthened within this subgroup.

**Conclusions:**

Overall, the results demonstrated the effectiveness of targeting inflammation as a means of improving mood in SCI, with potential mechanisms relating to the reduction in IL-1β and improvements in levels of neuroactive compounds related to the kynurenine pathway. Due to the limited sample size, results should be interpreted with caution; however, they are worthy of further examination due to the potential impact of inflammation on depression.

**Trial registration:**

ClinicalTrials.gov ID: NCT02099890.

## Background

Individuals with major depressive disorder (MDD) are commonly reported to demonstrate immune dysfunction in the form of chronic inflammation, and likewise, individuals with chronic inflammatory disorders are more prone to MDD [1–3]. Such a state has been proposed to be a contributing factor to symptoms of depression due to the complex bidirectional communicatory pathways between various systems of the body. In this respect, chronic inflammation may impact the endocrine and nervous systems causing respective imbalances in critical hormones and neuroactive compounds which may ultimately influence behavior and contribute to depressive symptoms [4].

Various pro-inflammatory mediators possess the ability to influence the function of transporters, enzymes, and receptors both in the periphery and within the brain. Certain pro-inflammatory cytokines are able to travel from the periphery to the brain via both active and passive mechanisms [5, 6]. Once in the brain, pro-inflammatory cytokines such as IL-1β (interleukin-1 beta) and TNF-α (tumor necrosis factor alpha) can alter extra-cellular concentrations of serotonin (5-HT) by upregulating corresponding transporters (SERT) [7–9]. In this way, such pro-inflammatory cytokines have the ability to directly reduce 5-HT levels within the brain which are associated with symptoms of depression.

**Fig. 1:**
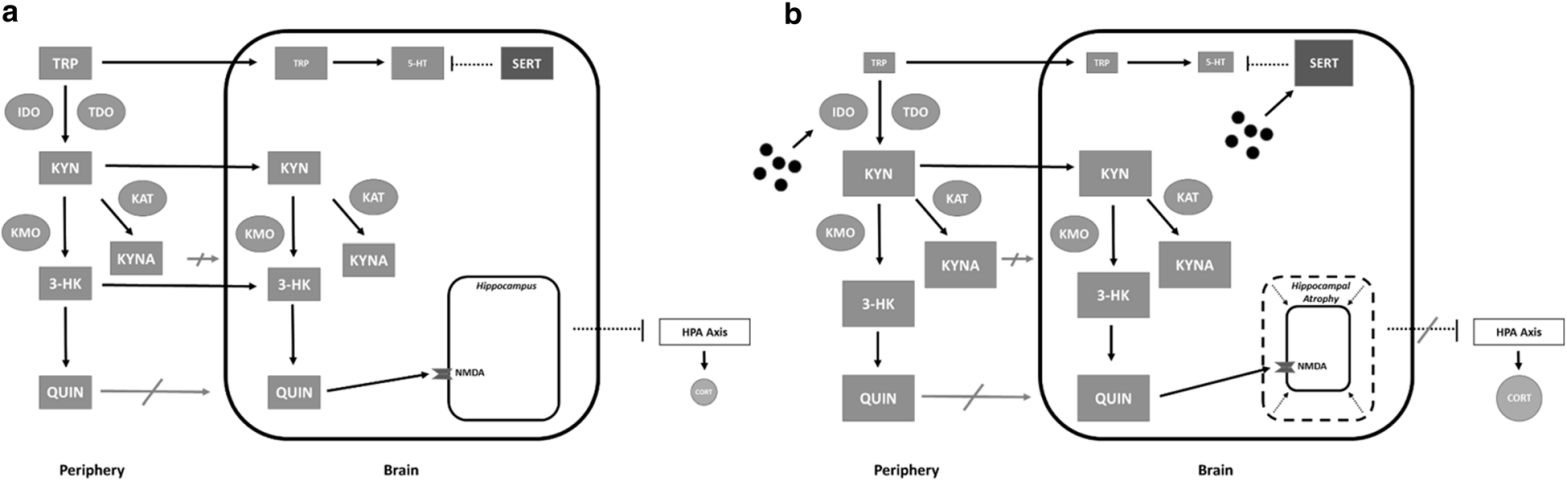
Tryptophan Metabolism along the kynurenine pathway. **a** The kynurenine pathway within the periphery and brain under healthy conditions. Proper enzyme regulation results in a healthy balance of TRP and TRP metabolites. As a result, adequate levels of TRP are available for 5-HT synthesis and properly regulated SERT proteins ensure that extra-cellular concentrations of 5-HT are not depleted. Healthy levels of TRP metabolites within the brain (e.g., QUIN) ensure normal activation of NMDA receptors thereby maintaining healthy hippocampal function and proper regulation of the HPA axis. **b** The kynurenine pathway within the periphery and brain in a state of chronic inflammation. Elevated levels of pro-inflammatory cytokines cause upregulation of enzymes such as IDO thereby resulting in an increased breakdown of TRP and production of TRP metabolites. As a result, reduced levels of TRP are available for the synthesis of 5-HT. Pro-inflammatory cytokines also upregulate SERT proteins causing further depletion of extra-cellular 5-HT. Elevated levels of TRP metabolites within the brain such as QUIN (potent NMDA agonist) result in over-activation of NMDA receptors, neuronal damage, and potential atrophy. This may also lead to a loss of inhibition of the HPA axis and excessive production of stress hormones

Peripherally, pro-inflammatory cytokines can influence the regulation of enzymes from critical metabolic pathways and induce imbalances in key mood-altering neuroactive compounds. The kynurenine pathway is one such pathway which must be strictly regulated due to its role as the primary route for tryptophan (TRP) degradation. Approximately 95 % of this critical 5-HT precursor is metabolized along this pathway, meaning a cytokine-induced upregulation of TRP degradation may ultimately result in the reduction in 5-HT levels within the brain [10, 11]. In the same respect, the increased degradation of TRP results in elevated levels of TRP metabolites. Certain TRP metabolites such as kynurenine (KYN) are BBB (blood brain barrier) transportable and have also been shown to contribute to symptoms of depression when at elevated concentrations within the brain (see Fig. 1).

A low-grade chronic inflammatory state is very commonly reported following spinal cord injury (SCI) as characterized by elevated levels of circulating pro-inflammatory mediators [12–16]. This state of immune dysfunction can be attributed to a variety of factors ranging from the loss of autonomic innervation of lymphoid organs, endocrine dysfunction, metabolic disorders, and a heightened risk for secondary health complications [17]. This population is also far more prone to depression with rates reaching up to five times (20–40 %) that of healthy, able-bodied individuals [18, 19]. Suicide rates are also far more prevalent among this population with rates ranging from three to five times greater than healthy able-bodied individuals [20, 21]. Provided that traditional pharmaceutical treatments for depression, such as selective serotonin reuptake inhibitors (SSRIs), have commonly reported side effects [22], prove ineffective in approximately 30 % of patients [23], and correspond to high rates of relapse [24], alternative treatments are needed.

The link between inflammation and depression may provide the opportunity to treat the latter by targeting the immune system via anti-inflammatory strategies. As there is a need for drastic changes in dietary intake following paralysis, and these changes are typically not met, the eating habits of individuals with SCI commonly contribute to a state of chronic inflammation. This provides the opportunity to reduce inflammation via the implementation of a strict anti-inflammatory diet for the purposes of assessing the influence of reduced inflammatory mediators on mood following SCI. It was hypothesized that reducing inflammation would lead to corresponding changes in neuroactive compounds and improvements in mood and depressive symptoms.

## Methods

### Study design and participants

The study was a randomized, parallel-group clinical trial. Participant recruitment occurred between September and November 2014. The study intervention was 12 weeks and included testing at baseline, 1 and 3 months. Participants with various levels and severities of SCI were recruited for participation in the study. Additional inclusion criteria included (1) over the age of 18, (2) SCI of any level or severity (American Spinal Injury Association A-D), and (3) at least 2 years post injury. Exclusion criteria included (1) any contraindications to supplements provided in the study, (2) unstable dosage of any anti-depressive medications, (3) an unstable medical condition within 2 weeks prior to intervention, (4) pregnancy, and (5) breastfeeding. Participant characteristics are shown in Table 1. Twenty individuals (10 male, 10 female; age 48.7 ± 13.9 years) with chronic (4–37 years post injury) SCI (C2-L4; ASIA Impairment Scale [AIS] A-D) were recruited for participation in the study. Twelve participants were randomly allocated to the treatment group, and were placed on the 12-week anti-inflammatory diet intervention, while eight were allocated to the control group and received no intervention. Informed consent was obtained from all participants. The study was registered as a clinical trial (clinicaltrials.gov identifier: NCT02099890) and received ethical approval from the Brock University Research Ethics Board as well as the Natural Health Products Directorate of Canada. All data was collected on-site at Brock University and the Brock-Niagara Center for Health and Well-being.

**Table 1:** Participant characteristics

Participant	Sex	Age	AIS score	Level of injury	Time since injury (years)
Treatment					
1	F	44	D	C5	10
2	M	58	B	T10	4
3	F	62	D	L3	4
4	F	37	A	T3	19
5	M	22	A	C7	5
6	M	67	C	C2	4
7	M	66	D	C5	6
8	F	44	A	C7	9
9	F	65	D	T6	4
10	F	64	D	C3	37
11	M	45	A	T6	28
12	M	37	C	C4	23
Control					
13	F	30	B	C5	6
14	F	63	D	L4	2
15	M	42	A	C5	6
16	F	58	D	C5	33
17	M	59	D	T4	4
18	F	33	A	T1	17
19	M	41	C	C4	22
20	M	36	A	C5	19

*AIS* ASIA (American Spinal Injury Association) Impairment Scale

### Randomization

Randomization was computer-generated by the primary investigator and stratified by participant gender and age using permuted blocks of 2 (male/female) and blocks of 3 (<40, 40–60, >60 year). Randomization was 3:2 to either the anti-inflammatory diet vs. control.

### Anti-inflammatory diet intervention

The anti-inflammatory diet intervention focused on the elimination of common food intolerances and inflammation-inducing foods, as well as the introduction of foods and supplements with established anti-inflammatory properties. Examples of foods removed from the diet included those with high glycemic indices (such as refined wheat products and refined sugars), common intolerances such as cow’s milk and foods which negatively influence cardiovascular health such as hydrogenated oils. Participants also consumed daily supplements with established anti-inflammatory benefits. Omega-3 (Now Ultra omega-3) was taken in softgel form, containing 500 mg EPA and 250 mg DHA, at a dosage of three per day. Chlorella (Now chlorella) was taken in pill form, containing 1000 mg, at a dosage of six per day. Antioxidants (CanPrev antioxidant network) were taken in pill form, containing 100 mg coenzyme Q10, 200 mg n-acetyl-cysteine, 150 mg mixed tocopherols, 100 mg DL alpha lipoic acid, 60 mg green tea extract, 5.5 mg zinc, and 100 μg selenium, at a dosage of two per day. Curcumin (AOR Inflanox) was taken in pill form, containing 400 mg, at a dosage of three per day. A vegetable-based protein powder (Progressive Vegessential) containing 27 g of protein was taken at a dosage of one scoop each morning.

Both the treatment group and control group were asked to complete a detailed diet record for 7 days at baseline, as well as 3 days at 1, 2, and 3 months in order to establish baseline eating habits and assess compliance throughout the intervention. Food intake was assessed using The Food Processor (ESHA Inc. 2014, version 10.14.2, Salem, OR). Compliance to the specific anti-inflammatory diet was also assessed by a detailed analysis of all diet records. Each food item was categorized as either “food to consume”, “food to avoid”, or “neutral food” based on the parameters of the diet participants were instructed to follow. Food was also categorized into servings in accordance with Canada’s Food Guide. Therefore, compliance scores were based on standard servings of foods subjects were instructed to eat vs. foods they were instructed to avoid. To account for differences in total energy intake, compliance scores were expressed as a ratio of the servings of foods to consume over the total servings of food (avoid + consume) multiplied by 100. The percent compliance was then generated.

The treatment group then underwent an information seminar which explained the diet program followed by a one-on-one consultation with nutritionists during which their diet records were reviewed in detail, and necessary changes were discussed. Participants received information regarding foods to eat and avoid, a supplement intake schedule, and a list of approved recipes. Participants in the treatment group received support via weekly phone calls from members of our research team as well as a monitored online support group whereby participants could share recipes and experiences with one another. Participants in the control group were asked to maintain their current diets throughout the duration of the study.

### Measurement of serum inflammatory markers and amino acids

Blood draws (20 ml) were taken from the antecubital vein of each participant at 1 p.m. at each of the three testing sessions (baseline, 1 and 3 months). Following extraction, the whole blood was allowed to clot for 30 min followed by centrifugation at 1000 ×*g* for 15 min. Serum was extracted and immediately stored at −80 °C until later analysis. Inflammatory mediators of interest included the pro-inflammatory cytokines IL-2, IL-1β, IL-6, TNF-α, IFN-γ, the acute phase protein CRP, as well as the anti-inflammatory cytokines IL-4, IL-10, and IL-1RA. Amino acids of interest included tryptophan, phenylalanine, tyrosine, and branched-chain amino acids (valine, leucine, isoleucine) for the assessment of the TRP/LNAA ratio. Phenylalanine and tyrosine were also quantified for the assessment of the PHE/TYR ratio which provides an indication of phenylalanine (4)-hydroxylase activity. Chronic inflammation has been shown to impair the activity, this enzyme thereby causing increases in PHE and an increase in the PHE/TYR, which provides some indication of the inflammatory state [25]. Kynurenine levels were also analyzed for the assessment of the KYN/TRP ratio. Analysis of pro- and anti-inflammatory cytokines was performed in triplicate via the Magpix Multiplex system (EMD Millipore, MA, USA.) and analyzed using Luminex software. CRP was analyzed in triplicate and quantified via enzyme-linked immunosorbent assay (R&D systems, Minneapolis, USA). TRP, Phe, TYR, BCAA, and KYN were analyzed in triplicate and quantified via enzyme-linked immunosorbent assay (Immunodiagnostik, Bensheim, Germany; Labor Diagnostika, Nordhorn, Germany).

### Assessment of mood

Participants completed the Center for Epidemiological Studies Depression Scale (CES-D) at each of the three testing sessions, as a means of assessing symptoms of depression. The questionnaire consisted of 20 items related to depression, and participants were asked to rate how often they experienced each item over the previous 7-day period. Ratings were based on a 4-point scale including “rarely or none of the time” (less than 1 day), “some or a little of the time” (1–2 days), “occasionally or a moderate amount of the time” (3–4 days), or “most or all of the time” (5–7 days). Points for each item ranged from 0 to 3 depending on frequency, and each item was summed for a total score ranging from 0 to 60 with higher scores indicating the presence of more symptomatology. A score of 16 points or greater is considered depressed.

### Statistical analysis

Two-way (group × time) repeated measure ANOVA was performed to investigate possible changes in inflammatory mediators, amino acids, and CES-D scores across three testing sessions. Post hoc analyses were used as needed to compare means when significant group × time interactions were found. These data are expressed as means ± standard deviations. Correlations between changes in inflammatory mediators, amino acids, and CES-D scores were assessed by means of Pearson’s *r* correlation. A mediation analysis was performed to assess whether the relationship between the change in the KYN/TRP ratio and the change in CES-D score was mediated by the change in IL-1β. This analysis consisted of four steps of testing: (1) the association of the independent variable (∆KYN/TRP ratio) with the outcome variable (∆CES-D score), (2) the association of the independent variable with the mediator variable (∆IL-1β), (3) the association of the mediator variable with the outcome variable after controlling for independent variable, and (4) whether the effect of the independent variable on the dependent variable was reduced when the mediator was included in the model. Differences in IL-1β concentrations for subgroup analysis were assessed using the Student *t* test. Statistical significance was set at *p* ≤ 0.05 for all tests.

## Results

All participants from both the treatment and control group completed the entire 3-month duration of the study and were included in the analysis. No adverse events were reported. The participants’ overall compliance to the diet was assessed based on the average of the three diet records during the study (1, 2, and 3 months). One participant completed all three testing sessions but failed to produce the 2- and 3-month diet record. This participant had a dietary compliance over the first month of 92 %. All other participants completed each of the required diet records, and overall compliance ranged from 70 to 100 %, with a mean compliance of 89 %. A detailed analysis regarding specific diet adherence data will be presented elsewhere.

### All participants

#### Change in CES-D scores

Changes in CES-D scores are shown in Table 2. There was a significant group × time interaction for CES-D scores (*p* = 0.01, Cohen’s *d* = 1.07). Post hoc analysis showed a significant reduction in CES-D scores in the treatment group from both baseline to 1 month, as well as from baseline to 3 months (*p* = 0.01 and *p* < 0.01, respectively). Post hoc analysis showed a significant reduction in CES-D score in the control group from baseline to 1 month (*p* = 0.03) but no significant change from baseline to 3 months (*p* = 0.74).

**Table 2:** Change in CES-D score and serum amino acids

	Treatment (*n* = 12)			Control (*n* = 8)		
	Baseline	1 month	3 months	Baseline	1 month	3 months
CES-D score***	14.5 ± 10.7	6.8 ± 5.6**	6.5 ± 5.0**	13.9 ± 12.0	11.3 ± 10.3*	14.6 ± 13.6
TRP (μmol/L)	89.2 ± 19.4	87.6 ± 28.8	90.7 ± 26.7	107.8 ± 28.1	105.9 ± 20.3	102.6 ± 14.4
Tyrosine (μmol/L)	48.2 ± 17.7	50.5 ± 15.1	41.5 ± 15.2	59.4 ± 17.6	53.7 ± 15.7	62.2 ± 18.3
Phenylalanine (μmol/L)	36.6 ± 13.6	36.8 ± 13.6	33.6 ± 14.8	42.0 ± 16.6	40.8 ± 18.2	46.2 ± 23.4
BCAA (μmol/L)	700.2 ± 462.4	651.3 ± 351.9	467.9 ± 198.5	449.7 ± 105.5	472.2 ± 112.1	534.3 ± 159.2
LNAA (μmol/L)	785.0 ± 468.2	738.6 ± 352.3	543.1 ± 217.3	551.2 ± 108.7	566.8 ± 123.8	642.7 ± 190.3
TRP/LNAA***	146.9 ± 71.3	133.6 ± 52.3	188.7 ± 90.4	206.3 ± 79.6	194.0 ± 51.2	171.2 ± 49.8
KYN (μmol/L)	2.3 ± 0.7	2.3 ± 0.6	2.1 ± 0.4	1.7 ± 0.4	2.1 ± 0.3	2.1 ± 0.4
PHE/TYR	786.8 ± 232.2	738.1 ± 229.5	809.5 ± 180.4	746.6 ± 247.1	751.08 ± 219.1	732.78 ± 267.2
KYN/TRP	27.0 ± 11.6	28.5 ± 10.9	24.0 ± 6.6	16.8 ± 4.9	20.3 ± 4.7	20.6 ± 6.0

LNAA (large neutral amino acids) is the sum of tyrosine, phenylalanine, and BCAA; TRP/LNAA ratio is calculated as TRP/LNAA × 1000; KYN/TRP ratio is calculated as KYN/TRP × 1000; and PHE/TYR ratio is calculated as PHE/TYR × 1000

All results are shown as mean ± SD

*Significantly different from baseline with *p* value ≤0.05

**Significantly different from baseline with *p* value ≤0.01

***Significant group × time interaction with *p* value ≤0.05

#### Change in serum amino acids

Changes in serum amino acids are shown in Table 2. There was a significant group × time interaction for the TRP/LNAA ratio (*p* = 0.04, Cohen’s *d* = 0.90). Post hoc analysis showed a trend toward a reduction in TRP/LNAA in the treatment group from baseline to 3 months (*p* = 0.06). Post hoc analysis showed no change in the control group at any time point. There was no significant group × time interaction for the KYN/TRP ratio (*p* = 0.32, Cohen’s *d* = 0.51) or the PHE/TYR ratio (*p* = 0.435, Cohen’s *d* = 0.43). KYN showed a trend toward a group × time interaction (*p* = 0.06, Cohen’s *d* = 0.81).

#### Change in inflammatory mediators

Changes in serum levels of inflammatory mediators are shown in Table 3. When considering a composite score averaging pro-inflammatory cytokines, the two-way (group × time) ANOVA showed a significant interaction (*p* = 0.04, Cohen’s *d* = 1.02). Post hoc analysis showed trends toward reductions in the treatment group from both baseline to 1 month and baseline to 3 months (*p* = 0.08 and *p* = 0.10, respectively) and no significant change in the control group at any time point. When analyzing each cytokine separately, two-way ANOVA showed significant group × time interactions for IFN-γ (*p* = 0.03, Cohen’s *d* = 1.02) and IL-1β (*p* = 0.04, Cohen’s *d* = 0.87). Post hoc analysis showed trends toward reductions in the treatment group from both baseline to 1 month and baseline to 3 months for IFN-γ (*p* = 0.08 and *p* = 0.09, respectively) and no significant change in the control group at any time point. Post hoc analysis showed a significant reduction in IL-1β in the treatment group from baseline to 1 month (*p* = 0.04) and a trend from baseline to 3 months (*p* = 0.07) and no significant change in the control at any time point. Trends toward group × time interactions were observed for IL-6 (*p* = 0.10, Cohen’s *d* = 0.74), TNF-α (*p* = 0.10, Cohen’s *d* = 0.74), and IL-1RA (*p* = 0.07, Cohen’s *d* = 0.79).

**Table 3:** Change in inflammatory mediators

	Treatment (*n* = 12)			Control (*n* = 8)		
	Baseline	1 month	3 months	Baseline	1 month	3 months
Pro-inflammatory cytokines						
CRP (ng/ml)	4474.7 ± 3578.9	3822.6 ± 3749.4	2865.0 ± 2684.9	2388.1 ± 2928.1	3074.0 ± 3026.4	2458.8 ± 3678.9
IL-2 (pg/ml)	21.3 ± 51.2	15.1 ± 41.7	17.2 ± 42.1	1.7 ± 3.4	2.9 ± 3.6	2.3 ± 3.3
IL-6 (pg/ml)	13.9 ± 28.2	9.2 ± 21.3	9.5 ± 19.3	9.0 ± 10.5	13.8 ± 21.2	13.5 ± 21.9
IL-1β (pg/ml)**	0.9 ± 1.1	0.3 ± 0.3*	0.3 ± 0.2	0.3 ± 0.3	0.4 ± 0.5	0.3 ± 0.2
TNF-α (pg/ml)	12.5 ± 3.6	11.8 ± 5.5	11.2 ± 4.1	9.8 ± 3.9	11.3 ± 6.7	12.9 ± 10.3
IFN-γ (pg/ml)**	52.9 ± 94.0	31.9 ± 57.5	35.0 ± 68.4	28.1 ± 46.8	48.8 ± 84.6	49.6 ± 95.3
Anti-inflammatory cytokines						
IL-4 (pg/ml)	7.5 ± 20.8	12.4 ± 23.9	16.2 ± 38.4	19.8 ± 37.2	37.4 ± 83.8	23.8 ± 46.3
IL-10 (pg/ml)	6.5 ± 12.9	11.2 ± 29.7	9.3 ± 22.0	5.9 ± 14.4	5.7 ± 13.7	6.3 ± 14.6
IL-1RA (pg/ml)	33.1 ± 26.2	27.8 ± 18.6	26.3 ± 16.0	60.6 ± 66.6	77.2 ± 77.2	78.8 ± 105.8

Pro-inflammatory cytokines consist of a composite score averaging IL-2, IL-6, IL-1β, TNF-α, and IFN-γ; anti-inflammatory cytokines consist of a composite score averaging IL-4, IL-10, and IL-1RA

All results are shown as mean ± SD

*Significantly different from baseline with *p* value ≤0.05

**Significant group × time interaction with *p* value ≤0.05

### Correlational analysis

Pearson’s *r* correlation coefficients for amino acid, cytokine, and CES-D data are shown in Table 4. The change in IL-1β was significantly correlated with both the change in CES-D score (*r* = 0.740, *p* < 0.01) and the change in the KYN/TRP ratio (*r* = 0.536, *p* = 0.02). The change in the KYN/TRP ratio was also significantly correlated with the change in CES-D score (*r* = 0.586, *p* = 0.01). The relationship between the change in TRP/LNAA ratio and the change in CES-D score did not reach statistical significance (*r* = −0.378, *p* = 0.10), whereas the relationship between the change in the TRP metabolite KYN and the change in CES-D score did reach statistical significance (*r* = 0.705, *p* < 0.01).

**Table 4:** IL-1β, amino acid, and CES-D correlations

	∆CES-D	∆IL-1β	∆TRP/LNAA	∆KYN/TRP
All participants (*n* = 20)				
∆CES-D	–	–	–	–
∆IL-1β	0.740**	–	–	–
∆TRP/LNAA	−0.378	0.369	–	–
∆KYN/TRP	0.586**	0.536*	0.345	–
∆KYN	0.705**	0.657**	0.114	0.730**
Subgroup (CES-D > 16) (*n* = 8)				
∆CES-D	–	–	–	–
∆IL-1β	0.738*	–	–	–
∆TRP/LNAA	0.569	0.363	–	–
∆KYN/TRP	0.937**	0.792*	0.436	–
∆KYN	0.800**	0.825**	0.192	0.823**

*p ≤ 0.05; **p ≤ 0.01

### Mediation analysis

A mediation analysis was performed in order to test whether the change in IL-1β mediated the observed relationship between the change in KYN/TRP and the change in CES-D scores. In the first step of the mediation model, the association of the independent variable with the outcome variable was assessed. This regression of the change in the KYN/TRP ratio on the change in CES-D scores (ignoring the change in IL-1β) was significant, *b* = 0.586, *t*(18) = 3.07, *p* = 0.01. In step two, the association of the independent variable with the mediator variable was assessed. It was shown that the regression of the change in the KYN/TRP ratio on the mediator (change in IL-1 β) was also significant, *b* = 0.06, *t*(18) = 2.70, *p* = 0.02. In step three of the mediation process, the association of the mediator variable with the outcome variable after controlling for the independent variable was assessed. The regression of the change in IL-1β on the change in CES-D score (controlling for the change in the KYN/TRP ratio) was significant *b* = 5.34, *t*(17) = 3.30, *p* < 0.01. In the last step, we assessed whether the effect of the independent variable on the dependent variable was reduced when the mediator was included in the model. When controlling for the change in IL-1β, the change in the KYN/TRP ratio was not a significant predictor of the change in CES-D score, *b* = 0.25, *t*(17) = 1.45, *p* = 0.16. A Sobel test was conducted and found full mediation in the model (*z* = 2.04, *p* = 0.04). Therefore, it was found that the change in IL-1β fully mediated the relationship between the change in the KYN/TRP ratio and the change in CES-D scores.

### Subgroup analysis

A subgroup analysis was performed examining participants with CES-D scores greater than 16 (indicating depression) from both the treatment group (*n* = 5) and control group (*n* = 3). It was found that participants with CES-D scores indicating depression had concentrations of IL-1β which were 73 % higher compared to those with lower (<16) CES-D scores (*p* = 0.05). Pearson’s *r* correlation coefficients for amino acid, cytokine, and CES-D data are shown in Table 4. The significant relationship between the change in IL-1β and the change in CES-D remained in this subgroup (*r* = 0.738, *p* = 0.04), and the relationship between the change in IL-1β and the change in the KYN/TRP ratio became even stronger (*r* = 0.792, *p* = 0.02). The change in the KYN/TRP ratio and the change in CES-D was also very strongly correlated in this subgroup (*r* = 0.937, *p* < 0.01). Once again, the change in the TRP/LNAA and the change in CES-D were not found to be significantly correlated (*r* = 0.569, *p* = 0.11) while the change in KYN was strongly correlated with the change in CES-D score (*r* = 0.800, *p* = 0.01).

## Discussion

The present study successfully obtained reductions in inflammatory mediators, modifications in neuroactive compounds, and improvements in mood in individuals with SCI by means of dietary alterations. As evidence suggests that inflammation contributes to depression, and SCI is typically characterized by a state of chronic inflammation as well as a high prevalence of depression, it was hypothesized that reducing levels of inflammation in this population would result in both molecular changes and corresponding improvements in mood.

Mood was significantly improved in the treatment group as demonstrated by the significant reduction in CES-D scores. When assessing potential mechanisms for such changes, a significant group × time interaction for the TRP/LNAA ratio was found, and post hoc analysis revealed trends toward an increase in the treatment group at 1 and 3 months following baseline. TRP is a critical precursor for 5-HT synthesis in the brain and competes for transportation across the BBB against other LNAA via common transport mechanisms. As such, an increase in the TRP/LNAA ratio signifies heightened TRP availability and would be expected to relate to improvements in mood. However, the relationship between the change in the TRP/LNAA ratio and the change in CES-D scores did not reach statistical significance (*p* = 0.10).

A significant group × time interaction was found for both IFN-γ and IL-1β, and post hoc analysis showed trends toward reduction for IFN-γ at 1 and 3 months and a significant reduction for IL-1β at 1 month and trend toward reduction at 3 months. Trends toward group × time interactions were also found for IL-6, TNF-α, and IL-1RA. Of these changes, only the reduction in IL-1β was shown to significantly correlate with the reduction in CES-D score. As IL-1β is capable of gaining access to the brain via leaky sites at the circumventricular organs or by crossing the BBB or via specialized active transporters, it may be possible for it to impose a direct influence [5, 6]. Once in the brain, IL-1β has been shown to activate 5-HT transporters (SERT), thereby stimulating the reuptake of 5-HT from the synaptic cleft, leading to its functional depletion [7–9]. Although it was not possible to directly assess SERT or 5-HT levels within the brain, it is possible for IL-1β to have contributed to changes in mood by means of such direct mechanisms.

The change in IL-1β was also significantly correlated with the change in the KYN/TRP ratio. The KYN/TRP ratio can represent the activity of either indoleamine 2,3-dioxygenase (IDO) or tryptophan 2,3 dioxygenase (TDO). Both are critical enzymes of the kynurenine pathway responsible for the degradation of TRP into KYN. TDO is, however, mainly expressed in the liver and not regulated by the immune system [26]. IDO is an immunoregulated enzyme, and elevations in the KYN/TRP ratio have been previously shown to be significantly correlated with increases in IFN-γ activity [27] as well as elevations in neopterin; a molecule synthesized by macrophages upon stimulation by IFN-γ, an indicator of a pro-inflammatory status [28]. This strongly suggests that IDO is the main enzymatic contributor to changes in the KYN/TRP ratio in response to changes in inflammation. Additional pro-inflammatory cytokines, including IL-1β, have been shown to upregulate IDO activity, thereby increasing the rate of TRP metabolism and production of TRP metabolites [29]. This selective TRP metabolism can lead to reductions in the TRP/LNAA ratio thereby limiting TRP availability for the synthesis of 5-HT. Additionally, the excessive breakdown of TRP results in the increased production of TRP metabolites which has also been shown to play an important role in factors related to depression. As the change in the KYN/TRP ratio was also significantly correlated with the change in CES-D scores, it may be possible that IL-1β influenced depression via such IDO-related mechanisms. Based on these findings, a mediation analysis was performed to further address the extent to which the link between change in the KYN/TRP ratio and change in the CES-D scores were mediated by change in the IL-1β. The analysis suggested that the relationship between change in the KYN/TRP and change in the CES-D was fully mediated by change in the IL-1β.

In addition to the potential reduction in TRP availability as shown by the TRP/LNAA ratio, a cytokine-induced upregulation of IDO activity may result in elevations in TRP metabolites. Several TRP metabolites, including KYN, are BBB transportable and can be neurotoxic when at elevated concentrations within the brain. As in the periphery, once in the brain, KYN can be further metabolized along the kynurenine pathway into other metabolites which have been shown to influence depression. One such metabolite, quinolinic acid (QUIN), acts as a potent agonist of the N-methyl-D-aspartate (NMDA) receptor which is densely populated on the hippocampus and plays a critical role in synaptic plasticity. Elevated concentrations of QUIN may result in NMDA over-activity resulting in an increased calcium influx leading to corresponding neuronal damage and potential atrophy [30]. Smaller hippocampal volumes in individuals with MDD have been commonly reported [31–34]. As the hippocampus plays an important role concerning the inhibition of HPA axis, hippocampal atrophy may result in an upregulated HPA axis and excessive glucocorticoid production from the adrenal gland. Hyperactivity of the HPA axis and elevated levels of stress hormones have also been commonly reported in individuals with MDD (see Fig. 1) [35, 36].

Both the reduction in the TRP/LNAA ratio and increase in TRP metabolites have the potential to influence depression via the aforementioned mechanisms. However, the fact that the change in KYN was related to the change in CES-D scores (while the change in TRP/LNAA was not) may suggest that the production of TRP metabolites had a larger impact on such mood changes. Similar findings have been demonstrated by Sublette et al. 2011, whereby levels of KYN have been shown to be elevated in suicide attempters with MDD and correlated with attempt status, while TRP was not related. Further, Sublette et al. found a correlation between the cytokine activation marker neopterin and the KYN/TRP ratio suggesting that KYN production may have been influenced by inflammation [37].

Additional subgroup analysis on those with baseline CES-D scores greater than 16 (suggesting depression) further implicated a role for IL-1β in depression as levels were shown to be 73 % higher than in those with CES-D scores indicating a lack of depression. Further, all relationships between IL-1β, the KYN/TRP ratio and CES-D scores, were maintained or strengthened in this subgroup including a very strong relationship between the change in the KYN/TRP ratio and the change in CES-D scores. Despite the small sample size, it also worth noting that for the five participants in the treatment group who began with scores indicating depression, all five reached scores below 16 within the first month of the intervention.

The above mechanisms may explain the link between the dietary-induced reductions in inflammation and the improvements in mood as assessed by CES-D scores. SCI is associated with substantial alterations in nutrient requirements and energy demands as well as an increased risk for numerous metabolic disorders and acute infections. Appropriate dietary alterations are therefore a suitable method of reducing inflammation and have a number of established mechanisms of action ranging from altered gene transcription, changes in cell membrane composition, improved enzyme regulation, as well as improvements in metabolic health and body composition [38].

Common pharmaceutical treatment modalities for depression, such as selective serotonin reuptake inhibitors (SSRIs), are popular due to their ability to relieve symptoms in a relatively short period of time by acting on SERT proteins to help increase extra-cellular 5-HT levels. SSRI-use is, however, associated with a number of side effects [22] and only provides transient relief of symptoms as it does not target the etiological basis of the disorder. SSRIs have also been shown to be ineffective in approximately 30 % of patients [23] of whom a particularly elevated inflammatory state is typically reported [39, 40]. Additionally, of those who do respond to treatment, an estimated 20–80 % will relapse within the first 1–5 years following initial treatment [24]. As such, there is a clear need for a more sustainable, long-term treatment, free from the many side effects that coincide with prolonged use of traditional pharmaceutical treatments.

Whether anti-inflammatory strategies could be used as a stand-alone treatment or would need to be used in conjunction with other interventions has yet to be determined. Future, long-term studies with larger sample sizes and participants with various severities of depression will be needed. Several potential limitations of the current study should be noted. First, the study was only single blinded. While the examiner was blinded to group allocation during all blood analysis, participants were aware of their group assignment. Although placebo supplements could have been provided to the control group, it was not possible to adequately blind participants to all aspects of the diet. The treatment group underwent a highly restrictive diet while the control group was free to consume unhealthy foods making distinction between groups quite obvious. Second, it is not possible to elucidate the specific mechanisms related to the reductions in inflammation, nor is it possible to discern which aspects of the dietary intervention may have had the strongest effects. It will be necessary for future studies to examine aspects such as transcription factor activity and membrane composition in order to truly elucidate the means by which such interventions act to reduce inflammation and improve symptoms of neuropathic pain. In terms of generalizability, our sample was quite representative of the SCI population in Canada, in terms of age, level, and severity of injury [41]; however, the results may not necessarily be generalizable to all individuals with SCI with varying levels of inflammation and severities of depression (however, it is of interest that all participants with scores indicating depression improved to a score indicating a lack of depression in the current study). Finally, it is worth noting that utilizing an intervention which targets inflammation by such means as diet or exercise requires a commitment to a major lifestyle modification and may not provide the same immediacy of effects as traditional pharmaceuticals. Still, the current study showed strong compliance to the diet and achieved significant reductions in CES-D scores after only 1 month. Further, given the lack of side effects, and additional health benefits that coincide with healthy eating, such interventions may be a viable mode of treatment for this population and are worthy of further examination.

## Conclusions

The present study demonstrated that it was possible to improve mood in individuals with SCI by means of reducing inflammation. Such improvements may relate to positive changes in neuroactive compounds of the kynurenine pathway mediated by the significant reduction achieved in the pro-inflammatory cytokine IL-1β. These results suggest a potential role for anti-inflammatory interventions in the treatment of depression in spinal cord-injured individuals. Due to the limited sample size of the present study, the results should be interpreted with caution. However, this influence is worthy of further examination in future larger-scale studies, as it may help to reduce the reliance on traditional pharmaceuticals by complementing current treatments or by providing a safe and sustainable treatment alternative.
